# Beyond mild, moderate, and severe traumatic brain injury: modelling severity from clinical, neuroimaging, and blood-based indicators

**DOI:** 10.1016/j.ebiom.2025.106001

**Published:** 2025-11-04

**Authors:** Lindsay D. Nelson, Brooke E. Magnus, John K. Yue, Steve Balsis, Christopher J. Patrick, Nancy Temkin, Esther L. Yuh, Ramon Diaz-Arrastia, Ehri Ryu, Andrew I.R. Maas, David K. Menon, Lindsay Wilson, Geoffrey T. Manley, the TRACK-TBI Investigators, Ramesh Grandhi, C. Dirk Keene, Christopher Madden, Michael McCrea, Randall Merchant, Pratik Mukherjee, Laura B. Ngwenya, Ava Puccio, Claudia Robertson, David Schnyer, Sabrina R. Taylor, Mary Vassar, CENTER-TBI Participants and Investigators, Cecilia Ackerlund, Hadie Adams, Krisztina Amrein, Nada Andelic, Lasse Andreassen, Audny Anke, Anna Antoni, Gérard Audibert, Philippe Azouvi, Maria Luisa Azzolini, Ronald Bartels, Pál Barzó, Romuald Beauvais, Ronny Beer, Bo-Michael Bellander, Antonio Belli, Habib Benali, Maurizio Berardino, Luigi Beretta, Morten Blaabjerg, Peter Bragge, Alexandra Brazinova, Vibeke Brinck, Joanne Brooker, Camilla Brorsson, Andras Buki, Monika Bullinger, Manuel Cabeleira, Alessio Caccioppola, Emiliana Calappi, Maria Rosa Calvi, Peter Cameron, Guillermo Carbayo Lozano, Marco Carbonara, Ana M. Castaño-León, Simona Cavallo, Giorgio Chevallard, Arturo Chieregato, Giuseppe Citerio, Hans Clusmann, Mark Steven Coburn, Jonathan Coles, Jamie D. Cooper, Marta Correia, Amra Čović, Nicola Curry, Endre Czeiter, Marek Czosnyka, Claire Dahyot-Fizelier, Paul Dark, Helen Dawes, Véronique De Keyser, Vincent Degos, Francesco Della Corte, Hugo den Boogert, Bart Depreitere, Đula Đilvesi, Abhishek Dixit, Emma Donoghue, Jens Dreier, Guy-Loup Dulière, Ari Ercole, Patrick Esser, Erzsébet Ezer, Martin Fabricius, Valery L. Feigin, Kelly Foks, Shirin Frisvold, Alex Furmanov, Pablo Gagliardo, Damien Galanaud, Dashiell Gantner, Guoyi Gao, Pradeep George, Alexandre Ghuysen, Lelde Giga, Ben Glocker, Jagoš Golubović, Pedro A. Gomez, Johannes Gratz, Benjamin Gravesteijn, Francesca Grossi, Russell L. Gruen, Deepak Gupta, Juanita A. Haagsma, Iain Haitsma, Raimund Helbok, Eirik Helseth, Lindsay Horton, Jilske Huijben, Peter J. Hutchinson, Bram Jacobs, Stefan Jankowski, Mike Jarrett, Ji-yao Jiang, Faye Johnson, Kelly Jones, Mart Kals, Mladen Karan, Angelos G. Kolias, Erwin Kompanje, Daniel Kondziella, Lars-Owe Koskinen, Noémi Kovács, Ana Kowark, Alfonso Lagares, Linda Lanyon, Steven Laureys, Fiona Lecky, Didier Ledoux, Rolf Lefering, Valerie Legrand, Aurelie Lejeune, Leon Levi, Roger Lightfoot, Hester Lingsma, Marc Maegele, Marek Majdan, Alex Manara, Hugues Maréchal, Costanza Martino, Julia Mattern, Charles McFadyen, Catherine McMahon, Béla Melegh, Tomas Menovsky, Ana Mikolic, Benoit Misset, Visakh Muraleedharan, Lynnette Murray, Ancuta Negru, David Nelson, Virginia Newcombe, Daan Nieboer, József Nyirádi, Matej Oresic, Fabrizio Ortolano, Olubukola Otesile, Aarno Palotie, Paul M. Parizel, Jean-François Payen, Natascha Perera, Vincent Perlbarg, Paolo Persona, Wilco Peul, Anna Piippo-Karjalainen, Matti Pirinen, Dana Pisica, Horia Ples, Suzanne Polinder, Inigo Pomposo, Jussi P. Posti, Louis Puybasset, Andreea Rădoi, Arminas Ragauskas, Rahul Raj, Malinka Rambadagalla, Veronika Rehorčíková, Isabel Retel Helmrich, Jonathan Rhodes, Sylvia Richardson, Sophie Richter, Samuli Ripatti, Saulius Rocka, Cecilie Roe, Olav Roise, Jeffrey Rosenfeld, Christina Rosenlund, Guy Rosenthal, Rolf Rossaint, Sandra Rossi, Daniel Rueckert, Martin Rusnák, Juan Sahuquillo, Oliver Sakowitz, Renan Sanchez-Porras, Janos Sandor, Nadine Schäfer, Silke Schmidt, Herbert Schoechl, Guus Schoonman, Rico Frederik Schou, Elisabeth Schwendenwein, Charlie Sewalt, Ranjit D. Singh, Toril Skandsen, Peter Smielewski, Abayomi Sorinola, Emmanuel Stamatakis, Simon Stanworth, Robert Stevens, William Stewart, Ewout W. Steyerberg, Nino Stocchetti, Nina Sundström, Riikka Takala, Viktória Tamás, Tomas Tamosuitis, Mark Steven Taylor, Braden Te Ao, Olli Tenovuo, Alice Theadom, Aurore Thibaut, Matt Thomas, Dick Tibboel, Marjolijn Timmers, Christos Tolias, Tony Trapani, Cristina Maria Tudora, Andreas Unterberg, Peter Vajkoczy, Egils Valeinis, Shirley Vallance, Zoltán Vámos, Mathieu van der Jagt, Joukje van der Naalt, Gregory Van der Steen, Jeroen T.J.M. van Dijck, Inge A. van Erp, Thomas A. van Essen, Wim Van Hecke, Caroline van Heugten, Dominique Van Praag, Ernest van Veen, Roel van Wijk, Thijs Vande Vyvere, Alessia Vargiolu, Emmanuel Vega, Kimberley Velt, Jan Verheyden, Paul M. Vespa, Anne Vik, Rimantas Vilcinis, Victor Volovici, Nicole von Steinbüchel, Daphne Voormolen, Peter Vulekovic, Daniel Whitehouse, Eveline Wiegers, Guy Williams, Stefan Wolf, Zhihui Yang, Peter Ylén, Alexander Younsi, Frederick A. Zeiler, Agate Ziverte, Tommaso Zoerle

**Affiliations:** a Departments of Neurosurgery & Neurology, Medical College of Wisconsin, Milwaukee, WI, USA; b Department of Psychology and Neuroscience, Boston College, Boston, MA, USA; c Department of Neurosurgery, University of California, San Francisco, CA, USA; d Department of Psychology, University of Massachusetts Lowell, Lowell, MA, USA; e Department of Psychology, Florida State University, Tallahassee, FL, USA; f Departments of Neurological Surgery and Biostatistics, University of Washington, Seattle, WA, USA; g Department of Radiology and Biomedical Imaging, University of California, San Francisco, USA; h Department of Neurology, University of Pennsylvania, Philadelphia, PA, USA; i Department of Neurosurgery, University of Antwerp; and Antwerp University Hospital, Edegem, Belgium; j Department of Medicine, University of Cambridge, Addenbrooke’s Hospital, Cambridge, United Kingdom; k Division of Psychology, School of Natural Sciences, University of Stirling, Stirling, United Kingdom

**Keywords:** Traumatic brain injury, Severity, Classification, Blood-based biomarkers, Neuroimaging, Item response theory

## Abstract

**Background:**

The conventional clinical approach to characterising traumatic brain injuries (TBIs) as mild, moderate, or severe using the Glasgow Coma Scale (GCS) total score has well-known limitations, prompting calls for more sophisticated strategies.

**Methods:**

We used item response theory (IRT) to develop a new method for quantifying TBI severity using 24 clinical, head computed tomography, and blood-based biomarker variables familiar to clinicians and researchers. IRT uses individuals’ response patterns across indicators to estimate relationships between the indicators and a latent continuum of TBI severity. Model parameters were used to assign severity scores in two large cohorts, and associations with traditional GCS categories and 6-month functional outcomes (Glasgow Outcome Scale-Extended [GOSE]) were tested with correlational and logistic regression analyses.

**Findings:**

In the prospective Transforming Research and Clinical Knowledge in TBI (TRACK-TBI) cohort (*N* = 2545), modelling showed the 24 indicators index a common latent continuum of TBI severity. IRT enabled us to identify the relative contribution of these features to estimate an individual's TBI severity. Finally, within both the TRACK-TBI derivation sample and an external validation sample (Collaborative European NeuroTrauma Effectiveness Research in TBI [CENTER-TBI]), TBI severity scores generated using this novel IRT-based method incrementally predicted functional (GOSE) outcome better than classic clinical (mild, moderate, severe) or International Mission for Prognosis and Analysis of Clinical Trials in TBI (IMPACT) classification methods.

**Interpretation:**

Our findings directly inform ongoing international efforts to refine and deploy new pragmatic, empirically-supported strategies for characterising TBI, while illustrating a strategy that may be useful to improve staging systems for other diseases.

**Funding:**

This secondary analysis project was funded by the U.S. National Institute of Neurological Disorders and Stroke (Grant No. R01 NS110856).

**Research in context**

**Evidence before this study**

The most commonly used method for classifying traumatic brain injury (TBI) severity is to categorise the Glasgow Coma Scale total score into mild (13–15), moderate (9–12), and severe (3–8) levels. This method is well-recognised to be problematic, because it does not adequately reflect the heterogeneity of TBI, is not sensitive to the underlying pathophysiology of injury, and because the labels (“mild,” “severe”) can mislead patients and clinicians about individual patient prognoses and thereby undermine patient-centred care.

**Added value of this study**

This study uses a novel modelling approach to identify the relationship between diverse signs individually recognised to reflect acute TBI severity and to develop a new method for quantifying TBI severity. This provides a personalised, more precise method to score individuals' acute TBI severities, addressing calls to develop evidence-based approaches to improve the characterisation of TBI severity beyond mild, moderate, and severe TBI. The study's focus on clinically-recognised, validated individual indicators of TBI—i.e., clinical signs, neuroimaging findings, and blood-based biomarkers—improves understanding of TBI and sets the stage for future clinical adoption of improved TBI characterisation approaches.

**Implications of all the available evidence**

This work builds upon a wealth of recent work to validate neuroimaging and blood-based biomarkers of TBI, demonstrating that they can be used alongside traditional clinical signs to improve the characterisation of TBI severity. As a well-established modelling strategy that has been successfully applied to improve measurement of other conditions, the item response theory (IRT) approach taken shows promise to provide a flexible, rigorous approach to refining the characterisation of TBI. Together, recent advances in TBI science and the findings of this study provider a concrete path toward improving the detection of TBI in healthcare settings, acute clinical decision making, outcome prediction, and more individualised, patient-centred care.

## Introduction

The Glasgow Coma Scale (GCS)^1^ was the first widely adopted method for estimating traumatic brain injury (TBI) severity. Soon after its introduction, clinicians and researchers began using it to classify injuries into the broad categories of mild (GCS 13–15), moderate (GCS 9–12), or severe (GCS 3–8) TBI.^2^, ^3^, ^4^, ^5^ This practice began in the early 1980s as a way to describe TBI subpopulations and rapidly became commonplace,^3^^,^^4^ with later approaches evolving to additionally consider indicators such as duration of posttraumatic amnesia (PTA), duration of loss of consciousness (LOC), and presence versus absence of acute intracranial findings on clinical neuroimaging (generally computed tomography [CT]) scans; see Table 1 for common approaches for defining mild, moderate, and severe TBI).^2^^,^^6^, ^7^, ^8^, ^9^

**Table 1:** Commonly used systems for classifying TBI severity used to inform variables included in item response theory model.

GCS-based	**Mild**		**Moderate**	**Severe**
	GCS 13–15		GCS 9–12	GCS 3–8
U.S. Veteran's Affairs 3-group	**Mild**		**Moderate**	**Severe**
	GCS 13–15 LOC <30 min PTA <24 h AMS up to 24 h. CT−		GCS 9–12 LOC >30 min & <24 h. PTA >24 h & < 7 d AMS >24 h. CT− or CT+	GCS 3–8 LOC >24 h. PTA >7 d. AMS >24 h. CT− or CT+
4-group	**Uncomplicated mild**	**Complicated mild**	**Moderate**	**Severe**
	GCS 13–15 LOC <30 min PTA <24 h CT−	GCS 13–15 LOC <30 min PTA <24 h CT+	GCS 9–12 30 min < LOC < 24 h. 24 h < PTA < 7 d	GCS 3–8 LOC >24 h. PTA >7 d.

*Note*. The top-most GCS-based definition is perhaps the most widely used strategy to distinguish mild, moderate, and severe TBI.^2^, ^3^, ^4^ The U.S. Department of Defense/Department of Veteran's Affairs adheres to the middle definition,^6^ while the latter 4-group classification system employed Harvey Levin's work to distinguish complicated (CT+) from uncomplicated (CT−) mild TBI and has been widely used in the field of neuropsychology.^7^, ^8^, ^9^ If a patient meets criteria in more than one category of severity, the higher severity level is assigned. *Abbreviations*: AMS, alteration of consciousness/mental status; CT, head computed tomography scan (CT+ reflects the presence of acute intracranial findings, CT− reflects that acute intracranial findings are absent or the test was not performed); GCS, Glasgow Coma Scale score; LOC, loss of consciousness; PTA, posttraumatic amnesia; TBI, traumatic brain injury.

Although the GCS-based staging convention addresses clinical and research needs to characterise and communicate about severity, it has been criticised for its lack of precision and insensitivity to the heterogenous pathologies of TBI.^2^^,^^10^^,^^11^ The 3-category GCS approach, for example, labels over 90% of TBIs “mild,”^12^^,^^13^ which can be misleading given the varied, sometimes poor outcomes of this TBI subgroup.^10^^,^^14^^,^^15^ Besides loss of information in staging patients using GCS total scores,^2^ TBI severity classification approaches rely on clinical signs of altered consciousness that may be confounded by non-TBI factors common in patients with trauma, such as alcohol/substance intoxication, use of sedatives and analgesics, and extracranial injuries.^2^^,^^10^ For this reason, experts have called for the development of new ways to quantify TBI severity that would use not only traditional clinical signs but also more direct indicators of injury pathophysiology such as specific radiographic findings and biochemical measures (e.g., blood-based biomarkers).^11^^,^^16^ Yet current systems that incorporate objective brain injury markers do so by dichotomising head CT findings as positive or negative, conflating neuroradiologic findings with widely disparate, or even opposing, effects on long-term outcome. In particular, CT findings are commonly considered “positive” for acute intracranial injury due to epidural haematomas (EDH). However, large-scale studies of severe TBI have not found EDH to indicate poor long-term prognosis, whereas other imaging findings (e.g., subarachnoid haemorrhage [SAH], subdural haematoma [SDH]) are robustly associated with adverse long-term outcomes. It remains unclear how these types of neuroimaging findings should be used when quantifying TBI severity.^17^

To date, efforts to enhance TBI severity grading systems have been limited by a lack of large, well-characterised TBI samples and objective biomarkers suitable for clinical use to better detect underlying pathophysiology. Additionally, because TBI severity is reflected in indicators across measurement domains (e.g., clinical, neuroimaging, blood-based markers), tools are needed to empirically position diverse indicators along the underlying continuum of TBI severity. Recent large-scale prospective TBI studies provide invaluable data to cultivate new strategies for characterising TBI severity. Using the large prospective Transforming Research and Clinical Knowledge in TBI (TRACK-TBI) sample of United States (U.S.) Level I trauma centre patients, we developed a novel data-driven approach to characterise the broader spectrum of TBI severity.

We used a novel item response theory (IRT) approach to model the continuum of TBI severity from diverse clinical signs and objective injury-related biomarkers. IRT is a statistical framework suited to identify an individual's position along a continuum using indicators from differing measurement domains. Following recommendations that new TBI classification systems be pragmatic,^18^ analyses focused on classifying TBI severity using variables widely available in the acute care setting (e.g., GCS, head CT) or on the near horizon of clinical translation (blood-based biomarkers). In particular, the individual GCS components (eye, motor, verbal), LOC duration, PTA duration, pupil reactivity, and 13 specific CT findings (e.g., contusion, SDH) were incorporated into models with more precision than current grading systems, to emphasise pathoanatomic imaging features in classification, enable clearer differentiation among patients, and empirically determine the contributions of each indicator on the severity spectrum. Additionally, we incorporated several blood-based biomarkers to address calls for incorporating more biological markers into TBI severity grading.^11^ Their inclusion was justified by the near-term feasibility of employing them clinically (e.g., two markers—glial fibrillary acidic protein [GFAP] and ubiquitin C-terminal hydrolase [UCH-L1]—were already FDA- and EMA-approved for decisions about neuroimaging and a third—S100 calcium binding protein B [S100B]—was included in Scandinavian guidelines for managing GCS 14–15 TBI).^19^, ^20^, ^21^ Finally, we used the model to score individuals in the TRACK-TBI derivation sample and an external validation sample (Collaborative European NeuroTrauma Effectiveness Research in TBI [CENTER-TBI]) on the dimension of TBI severity in order to demonstrate the distribution and predictive value of novel TBI Severity IRT scores. Establishing the relationship between clinically relevant signs of TBI severity may corroborate what is known from clinical experience and studies of individual signs. By establishing that these clinical signs and biomarkers of TBI reflect a single underlying dimension of severity and locating them on that continuum, this study can advance understanding of the spectrum of severity while offering a quantitative tool for further developing and refining practical TBI severity grading approaches.

## Methods

### Study design and participants

The TRACK-TBI study is a prospective observational cohort study of 2545 participants with TBI diagnosis aged ≥17 years from 18 U.S. Level I trauma centres, enrolled between 2014 and 2018, all of whom were included in the current analysis (Table 2). Inclusion criteria were: enrolment within 24 h of injury, CT scan ordered for clinical care, documentation of TBI consistent with the American Congress of Rehabilitation Medicine definition (i.e., head trauma resulting in neuroimaging structural brain injury and/or evidence of alteration of consciousness), and adequate visual acuity and hearing to complete outcome examinations. Exclusion criteria were being pregnant, in police custody, or on psychiatric hold; history of debilitating mental or neurological disorders; non-English- and non-Spanish-speaking; penetrating TBI; non-survivable (moribund) trauma; severe polytrauma or medical comorbidities (e.g., end-stage cancer) that would interfere with follow-up and outcome assessment; and being in an interventional trial.

**Table 2:** Sample characteristics, n (%) or Median (IQR).

	TRACK-TBI derivation sample *N* = 2545	CENTER-TBI external validation sample *N* = 4500
Demographics		
Age, years	38 (26, 55)	50 (30, 66)
Sex^a^		
Female	783 (30.8%)	1483 (33.0%)
Male	1762 (69.2%)	3017 (67.0%)
Race^a^		
Asian	93 (3.7%)	75 (1.7%)
Black	406 (16.0%)	63 (1.4%)
White	1949 (76.6%)	4155 (92.3%)
Other/unknown	97 (3.8%)	207 (4.6%)
Ethnicity^a^		
Hispanic	516 (20.3%)	–
Non-Hispanic	1990 (78.2%)	–
Unknown	39 (1.5%)	–
Education, years	12 (12, 16)	13 (10, 16)
Health insurance		
Medicaid/uninsured	789 (31.0%)	–
Other insurance	1571 (61.7%)	–
Unknown	185 (7.3%)	–
Injury characteristics		
Admission GCS total score^a^	15 (14, 15)	15 (10, 15)
3–8, n (%)	360 (14.5%)	986 (22.8%)
9–12, n (%)	123 (5.0%)	389 (9.0%)
13–15, n (%)	1995 (80.5%)	2955 (68.3%)
Positive head CT^b^	1162 (47.8%)	2430 (59.8%)
Number nonreacting pupils^c^		
Zero	2072 (93.4%)	3802 (89.5%)
One	42 (1.9%)	164 (3.9%)
Two	105 (4.7%)	281 (6.6%)
Cause of injury		
Motor vehicle/traffic crash	1456 (57.2%)	1682 (37.4%)
Fall	673 (26.4%)	2021 (43.9%)
Assault/violence	168 (6.6%)	242 (5.4%)
Other/unknown	248 (9.7%)	555 (12.3%)
Level of care/Stratum^d^		
Emergency department	530 (20.8%)	847 (18.8%)
Inpatient floor	870 (34.2%)	1523 (33.8%)
Intensive care unit	1145 (45.0%)	2137 (47.4%)
Loss of consciousness duration^c^		
None	282 (15.8%)	–
<1 min.	336 (18.8%)	–
1–29 min.	828 (46.4%)	–
30–59 min.	61 (3.4%)	–
1–24 h	114 (6.4%)	–
24 h–7 days	94 (5.3%)	–
>7 days	70 (3.9%)	–
Posttraumatic amnesia duration^c^		
None	416 (23.8%)	–
<1 min.	98 (5.6%)	–
1–29 min.	494 (28.2%)	–
30–59 min.	138 (7.9%)	–
1–24 h	363 (20.7%)	–
24 h–7 days	121 (6.9%)	–
>7 days	121 (6.9%)	–

*Note*. Sample reflects all TRACK-TBI study participants who had traumatic brain injury and were at least 17 years old. *Abbreviations*: CT, computed tomography scan; CENTER-TBI, Collaborative European NeuroTrauma Effectiveness Research in Traumatic Brain Injury study (validation sample); GCS, Glasgow Coma Scale; min., minutes; TRACK-TBI, Transforming Research and Clinical knowledge in TBI study (derivation sample).

a The study did not record how biological sex, race, and ethnicity was collected.

b Positive findings included any acute intracranial haemorrhage (i.e., subdural haematoma, epidural haematoma, subarachnoid haemorrhage, contusion, intraventricular haemorrhage, small haemorrhages consistent with traumatic axonal injury, and intracerebral haematoma), any acute brain herniation (midline shift, partial or complete effacement of basal cisterns, any cerebellar herniation), acute infarct, and brain swelling or oedema).

c n = 77 missing Admission GCS; n = 110 missing Head CT outcome; n = 236 missing pupil reactivity; n = 760 missing loss of consciousness duration; n = 794 missing posttraumatic amnesia duration.

d Variable reflects the highest level of care in the TRACK-TBI sample, and the level of care at the time of enrolment in the CENTER-TBI sample.

The CENTER-TBI study was a prospective, longitudinal study that enrolled patients from 55 European trauma centres, with similar inclusion criteria and follow-up protocols as TRACK-TBI.^22^ Of the 4509 individuals enrolled, 4500 had data on the variables of interest to be included in the present study (Table 2).

### Ethics

The TRACK-TBI and CENTER-TBI studies obtained ethical approval at each enrolling site. TRACK-TBI completed informed consent with patients or legally authorised representatives whenever possible; some sites used an approved waiver of consent to enrol persons with impaired decision-making capacity.

CENTER-TBI obtained informed consent by the patients and/or the legal representatives/next of kins according to local legislations. The list of sites, Ethical Committees, approval numbers and approval dates can be found on the website: https://www.center-tbi.eu/project/ethical-approval. The CENTER-TBI study (EC grant 602150) has been conducted in accordance with all relevant laws of the EU if directly applicable or of direct effect and all relevant laws of the country where the Recruiting sites were located, including but not limited to, the relevant privacy and data protection laws and regulations (the “Privacy Law”), the relevant laws and regulations on the use of human materials, and all relevant guidance relating to clinical studies from time to time in force including, but not limited to, the ICH Harmonised Tripartite Guideline for Good Clinical Practice (CPMP/ICH/135/95) (“ICH GCP”) and the World Medical Association Declaration of Helsinki entitled “Ethical Principles for Medical Research Involving Human Subjects”. Informed Consent by the patients and/or the legal representative/next of kin was obtained, accordingly to the local legislations, for all patients recruited in the Core Dataset of CENTER-TBI and documented in the e-CRF.

### Procedures and variables for TRACK-TBI model development

#### TBI severity indicators

**Fig. 1:**
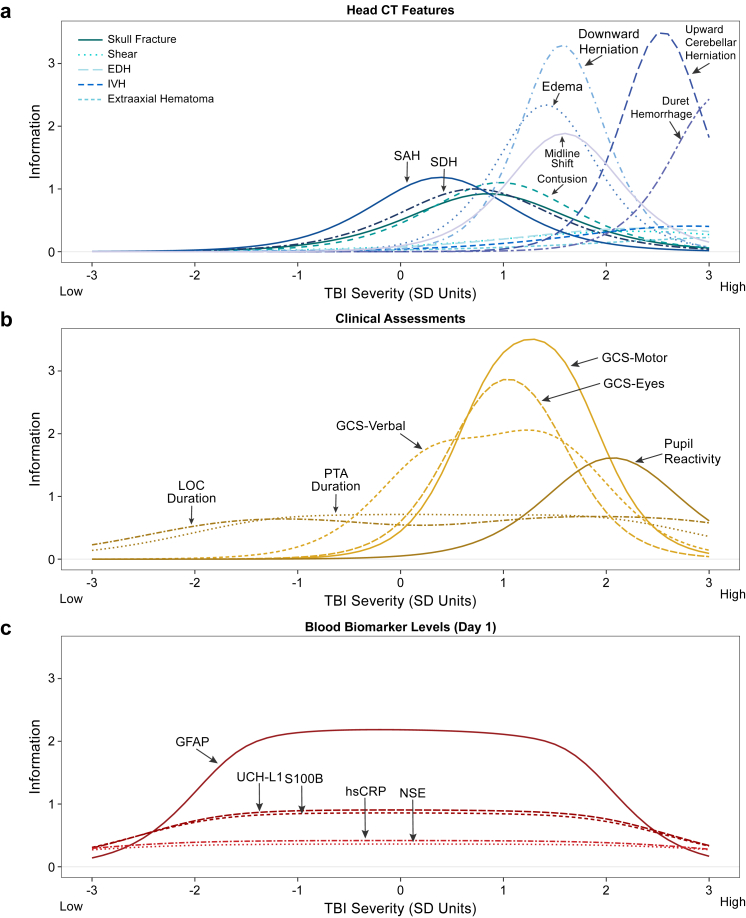
**Item information curves from a single item response theory (IRT) model of 24 TBI severity indicators, stratified by measurement domain for readability(a: brain imaging; b: clinical assessments; c: blood biomarkers)**. The model reflects all 2545 individuals with TBI age 17 or older in the TRACK-TBI study. The x-axis reflects the latent TBI severity spectrum modelled from the associations between the indicators using IRT. The y-axis reflects IRT *information*, which reflects the precision with which each variable can be used to measure individuals on the severity dimension, which can vary at different levels of severity. Higher information reflects lower standard errors to estimate individuals at a given level of severity. *Abbreviations*: CT, computed tomography; EDH, epidural haematoma; GCS, Glasgow Coma Scale; GFAP, glial fibrillary acidic protein; hsCRP, high-sensitivity C-reactive protein; IVH, intraventricular haemorrhage; NSE, neuron-specific enolase; LOC, loss of consciousness; PTA, posttraumatic amnesia; S100B, S100 calcium binding protein B; SAH, subarachnoid haemorrhage; SDH, subdural haematoma; TBI, traumatic brain injury; UCH-L1, ubiquitin C-terminal hydrolase.

Model development was performed on 24 indicators in the TRACK-TBI sample (named in Fig. 1; see also Supplementary Table S1). Admission GCS scores were extracted from medical records and coded separately for the eye (range 1–4), verbal (1–5), and motor (1–6) component scores. Untestable GCS codes (*N* = 6 eye, 187 verbal, 39 motor) were treated as missing to allow for GCS to be treated as an ordinal variable. Secondary analyses that included untestable GCS codes in IRT modelling (treating the GCS variables as nominal) supported their exclusion from IRT modelling (i.e., yielded unstable item parameter estimates and did not support these codes as indicating more severe injury). Duration of LOC and PTA were collected from medical records and/or participant (patient and/or legally authorised representative) interviews and coded in categories consistent with the National Institute of Neurological Disorders and Stroke TBI Common Data Elements (CDE).^23^ This information was collected via participant interview as soon as was feasible at one of the study visits (<24 h of injury; 2 weeks; and 3-, 6-, and 12-months post-injury).” Pupil reactivity (coded as number of nonreacting pupils; range 0–2), was included from participant medical records, given that it is commonly used clinically to detect more severe TBI and is a validated indicator of severity.^24^

Head CT scans performed on admission for clinical purposes were sent to a central imaging repository (Laboratory of Neuro Imaging, Los Angeles, CA, USA) and assessed by one board-certified neuroradiologist (ELY, who was blinded to injury and biomarker information) for findings consistent with the TBI CDE for Radiologic Imaging.^25^ Binary present/absent codes were used for each of 13 imaging findings associated with acute head trauma (SAH, acute SDH, midline shift, etc.).

#### Biospecimen collection and processing

Blood samples were collected in the hospital within 24 h of injury and then processed, aliquoted, and stored in a freezer within 2 h of collection. Analyses used data for the core set of biomarkers acquired for the full study sample: glial fibrillary acidic protein (GFAP), ubiquitin C-terminal hydrolase (UCH-L1), high-sensitivity C-reactive protein (hsCRP), S100 calcium binding protein B (S100B), and neuron-specific enolase (NSE). Coded blood samples were shipped from the study's central repository to site laboratories and analysed blinded to any clinical information. For biospecimen collection and processing procedures for the TRACK-TBI study, see: https://tracktbi.ucsf.edu/researchers. Plasma samples were analysed for GFAP and UCH-L1 at Abbott Laboratories (Abbott Park, IL, USA) on either the company's prototype point-of-care iSTAT Alinity System or the prototype core lab ARCHITECT platform. The measures were highly correlated and converted for analysis to iSTAT equivalent units for analysis.^26^ Analysis of hsCRP was carried out on serum samples by a laboratory at the University College of Dublin using the (Abbott ARCHITECT c8000, MULTIGENT CRP Vario assay) using the high-sensitivity method (CRP16).^27^ Analysis of S100B and NSE were conducted by a laboratory at the University College of Dublin using an electrochemiluminescence immunoassay (Elecsys® S100B; Roche Diagnostics, Penzberg, Germany) on an automated Cobas® system from Roche.^28^ Serum samples were thawed in batches at room temperature and centrifuged at 10 000 rcf for 10 min at 4 °C prior to testing in duplicate. The S100B assay is the trademarked assay used clinically in Europe for S100B (reportable range 0.005–39 μg/L; CV of 20%), which was optimised for serum. The reportable range of the NSE assay was 0.05–370 μg/L and CV was <20%.

#### Functional outcome

We evaluated incremental validity of our novel IRT-based TBI severity score for predicting functional outcome, as reflected by the Glasgow Outcome Scale-Extended (GOSE), over/above traditional classifications based on GCS total scores. The GOSE is an ordinal measure of global functional outcome that assigns one of 8 scores: 1 = Death; 2 = Vegetative State; 3 = Lower Severe Disability; 4 = Upper Severe Disability; 5 = Lower Moderate Disability; 6 = Upper Moderate Disability; 7 = Lower Good Recovery; 8 = Upper Good Recovery. Two GOSE scores were derived from structured interviews with patients and informants at 2 weeks and 6 months post-injury^29^—a GOSE-ALL score reflecting the overall change in functional independence due to all injury (TBI and extracranial) and a GOSE-TBI score, indicating the change in independence resulting solely from the TBI.^30^^,^^31^ To reflect qualitatively different functional outcomes and expected nonlinear associations between measures of TBI severity and GOSE outcomes,^32^ scores were dichotomised as death (GOSE = 1), unfavourable outcome (GOSE<5), and incomplete recovery (GOSE<8). Death and unfavourable outcome were defined to align with models used to develop International Mission for Prognosis and Analysis of Clinical Trials in TBI (IMPACT) scores, which were compared to IRT scores (see below).^24^ Complete recovery (GOSE = 8 versus <8) was included to align with studies of more mild injuries and provide a more comprehensive picture of the association between TBI severity and outcome.^33^^,^^34^

### CENTER-TBI validation sample

The CENTER-TBI study sample was used for external validation of the model developed in the TRACK-TBI sample. Details about the study, including biospecimen collection and processing, are available in prior publications.^35^ Based on the availability and equivalence of data across studies, 17 variables were used to estimate CENTER-TBI participants’ TBI Severity IRT scores using item parameters established in the TRACK-TBI sample. These included 9 CT (SAH, SDH, EDH, skull fracture, midline shift, shear, intraventricular haemorrhage, and extraaxial haematoma), 4 clinical (GCS eye, verbal, and motor scores; number of nonreactive pupils), and 4 biomarker variables (GFAP, UCH-L1, NSE, S100B; see also Supplementary Table S1. NSE and S100B were run on the same assay as TRACK-TBI. GFAP and UCH-L1 were run with a different assay (Quanterix Neuro 4-plex), but values converted to the scale used in TRACK-TBI using linking tables developed by running samples from TRACK-TBI on both assays.

### Statistics

Statistical analyses were conducted using R v.4.3.1,^36^ apart from initial factor analytic modelling, which was performed in Mplus v.8.10.^37^ IRT analyses were performed using the “mirt” package in R.^38^ We summarised the sample and TBI indicators of interest using descriptive statistics (frequencies/percentages; medians/interquartile ranges). Blood-based biomarker variables were categorised for all analyses into equal-sized groups, as needed for IRT modelling. Within the TRACK-TBI sample, we performed exploratory factor analysis (EFA) using diagonally weighted least squares estimation (WLSMV) to evaluate the key assumption underlying unidimensional IRT modelling: that the set of 24 TBI indicators (6 clinical signs, 13 acute head CT findings, and 5 blood-based biomarkers) predominantly indexed a single latent dimension. Meeting the *sufficient unidimensionality* criterion indicates that any multidimensional features within the data are small enough to not significantly bias parameter estimates in a unidimensional IRT model.^39^ Consistent with convention, we defined sufficient unidimensionality *a priori* as an EFA first-to-second eigenvalue ratio >4, and model fit statistics as follows: root mean square error of approximation (RMSEA) < 0.08, comparative fit index (CFI) > 0.90, and Tucker–Lewis Index (TLI) > 0.90.^39^, ^40^, ^41^, ^42^

We then fit a 2-parameter/graded response hybrid logistic unidimensional IRT model to the 24 indicators, which assumes a continuous latent dimension underlying the indicators and accommodates both binary items (presence/absence of each CT feature) and ordinal items (LOC and PTA duration, pupil reactivity, GCS scores, biomarkers). The model estimates two parameters per item, discrimination ($a_{j}$) and one or more threshold parameters ($b_{j}$). *Threshold* (or *difficulty*) reflects the location on the TBI severity continuum where a respondent has, for a binary indicator, a 0.5 probability of the indicator or, for a polytomous ordinal item, a 0.5 probability of displaying that category or a more severe one. *Discrimination* reflects the strength of the relationship between the item and the latent dimension; more discriminating items distinguish better between individuals who differ in TBI severity, especially at the threshold/difficulty level of the item.^39^ Analyses displayed the overall precision of each TBI indicator and their combined performance in a metric called *information*, which aggregates difficulty and discrimination and reflects the inverse of the standard error of measurement around estimates of the latent variable (TBI severity) across the continuum of severity.

The IRT model was identified by fixing the latent variable's mean to zero and its variance to one. The model used full-information maximum likelihood estimation, which provides robust estimates in the presence of ignorable missing data conditions (i.e., missing completely at random [MCAR] and missing at random [MAR] mechanisms) and allowed all enrolled participants to be included.^43^ Missing data rates varied by variable domain: 4.5–7.9% for CT features, 3.1–10.3% for GCS domains, 12.8% for pupil reactivity, 29.9–31.2% for LOC/PTA duration, and for 4.8%–17.9% blood-based biomarkers. Because having a head CT and providing a Day 1 research blood sample were inclusion criteria for study entry, missingness on these factors was likely MCAR, due to unsystematic protocol deviations and expected assay failure rates (e.g., due to sample mishandling and software/instrument issues).^44^ In support of this hypothesis, regression models predicting missingness on these features from other IRT model indicators not reveal any reliable patterns of missingness as a function of TBI severity. In contrast, clinical indicators were assumed to be MAR. For example, low-severity injuries may not prompt clinical documentation of TBI signs; in higher-severity injuries, signs may not be assessable due to TBI, extracranial injury, and associated medical treatments (e.g., sedation and mechanical ventilation).^2^ In support of MAR, we found associations between model indicators of more severe TBI and missingness on, for example, GCS Verbal, loss of consciousness, and posttraumatic amnesias.

The model yielded IRT-based TBI severity scores for each participant, which were submitted to further analyses to explore their potential validity and utility. Using the model parameters for each item established in the derivation sample, we also scored individuals from 17 available items in the CENTER-TBI sample to examine the validity of the model across both study samples.

In both study samples, we generated histograms to display the distribution of TBI Severity IRT scores; scatterplots to display the association between IRT scores and traditional TBI classifications (e.g., mild, moderate, severe); and fit separate sequential binary logistic regression models to examine the predictive value of TBI Severity IRT scores alone and their incremental predictive value over/above (i) traditional GCS-based mild, moderate, and severe TBI classification, and (ii) IMPACT scores.^24^

IMPACT scores were previously developed and cross-validated in large samples to prognosticate functional (GOSE) outcome in persons with GCS<13 TBI.^24^^,^^45^ Our logistic regression modelling separately tested IMPACT Core model (comprising age, GCS motor score, and pupil reactivity) and the IMPACT Extended model score (which adds to the Core model hypotension, hypoxia, and select head CT features), each of which produces separate scores for predicting mortality and unfavourable outcome (for details, see: http://www.tbi-impact.org/?p=impact/calc.) Nagelkerke *R*^2^ served as the primary indicator of model fit, and chi-square likelihood ratio tests used to evaluate the incremental predictive value of adding IRT scores to models incorporating traditional indicators of TBI severity.^46^

### Role of funders

The funders had no role in study design, data collection, data analyses, interpretation, or writing of this manuscript.

## Results

### Sample and IRT model fit

The TRACK-TBI sample (*N* = 2454) had a *Median* age = 38 (*IQR* = 26, 55) and was 69.2% male; the CENTER-TBI sample (*N* = 4500) had a *Median* age = 50 (*IQR* = 30, 66) and was 67.0% male. Both samples displayed diverse injury severity characteristics (Table 2). For example, 47.7% (TRACK-TBI) and 59.8% (CENTER-TBI) had acute intracranial findings on CT; 18.5% and 18.1%, respectively, were GCS 15 and showed no acute intracranial CT findings.

The EFA of the 24 acute TBI indicators supported proceeding with unidimensional IRT modelling and indicated that these diverse clinical signs, head CT findings, and blood-based biomarkers reflect a single common dimension, referred to henceforth as TBI severity (see Supplementary Figure S1; Supplementary Table S2).

### Characteristics of the IRT model

**Fig. 2:**
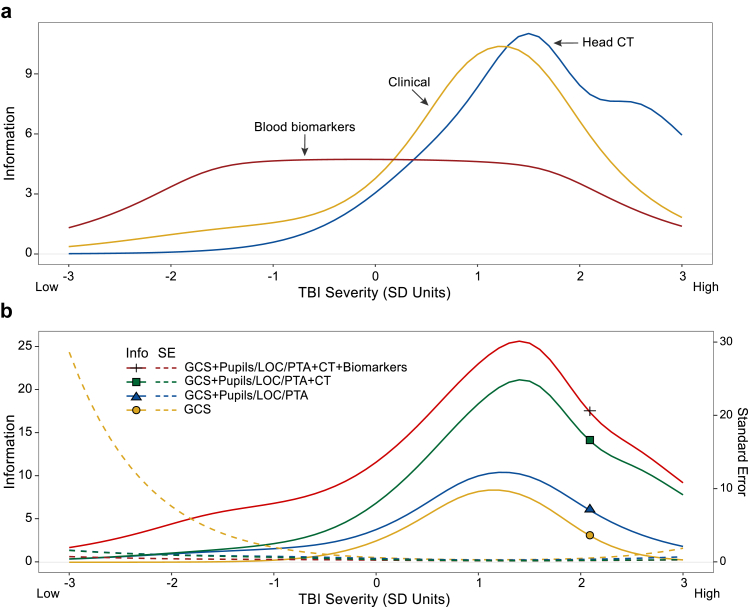
**Test information curves for TBI severity indicators grouped by measurement domains.** The model reflects all 2545 individuals with TBI age 17 or older in the TRACK-TBI study. **(a)** Test information curves for each measurement domain. Test information reflects the sum of item-level information (see Fig. 1) across all indicators (items) in each measurement domain. Higher information reflects greater measurement precision (i.e., lower standard error) in characterising and distinguishing persons at a given level of TBI severity. **(b)** Test information (solid lines) and associated standard errors (dashed lines) for increasingly complex sets of indicators, starting with GCS domain scores and adding, in order: pupil reactivity, LOC duration, and PTA duration; CT findings; and blood-based biomarkers. *Abbreviations*: EDH, epidural haematoma; GCS, Glasgow Coma Scale; GFAP, glial fibrillary acidic protein; hsCRP, high-sensitivity C-reactive protein; IVH, intraventricular haemorrhage; NSE, neuron-specific enolase; LOC, loss of consciousness; PTA, posttraumatic amnesia; S100B, S100 calcium binding protein B; SAH, subarachnoid haemorrhage; SDH, subdural haematoma; TBI, traumatic brain injury; UCH-L1, ubiquitin C-terminal hydrolase.

IRT model parameters are provided in Supplementary Table S3 (see Supplementary Material), which can be visualised as item (Fig. 1, Supplementary Figure S2) and test (Fig. 2a) information curves, with the latter reflecting the sum of information provided by items into each respective measurement domain (clinical, CT, and biomarker indicators). Fig. 2b displays the incremental improvements in test information (solid line) and reductions in standard error (dashed lines) achieved by adding to the GCS other types of data in the following order: CT; pupil reactivity, LOC duration, and PTA duration; and biomarkers. The information curves (which reflect a combination of the discrimination [*a*] and severity [*b*] IRT parameters) convey both where along the injury spectrum the indicators, or combination of indicators, fall (scaled in standard deviations) and which features are better- or worse-performing (i.e., higher or lower in height, respectively).

Looking at the domains as a whole, positive head CT findings indexed the upper half of the severity spectrum (*b* = 0.04–3.51 *SD*, where *b* reflects the levels of the latent TBI severity dimension where these indicators best distinguish individuals on severity), Within this upper half of severity, head CT features varied in the level of severity they indexed. For example, Duret haemorrhage—a brainstem haemorrhage associated with cerebral herniation—provided high information at the right-most portion of the spectrum (3.11 *SD*), indicating that it operates as a sensitive index of the most severe injuries. Evidence of herniation, oedema, and midline shift contributed information about mid-to-high range TBI severity (1.41–2.54 *SD*). Contusion, SAH, and SDH provided information about the mid-range of the severity spectrum (0.39–0.97 *SD*). Other features (e.g., EDH) provided little information (i.e., little ability to help differentiate individuals varying in severity). Skull fracture, although not technically a brain-injury-specific finding, fit within the model and indexed the latent spectrum similarly to SDH and contusion.

Similar to CT findings, clinical signs of GCS domain scores and pupil reactivity contributed to indexing injuries in the upper half of the severity spectrum (*b* = 0.34–2.17). While all GCS domains indexed a similar level of severity, the motor score displayed the highest information (precision). LOC duration (*b* = −1.65 to 2.84), PTA duration (*b* = −1.24 to 2.06), and blood-based biomarkers (*b* = −2.48 to 2.51) provided information across a wide spectrum of severity that included lower-severity injuries. Among the blood-based biomarkers, GFAP performed best to characterise severity. Within the lower half of the severity spectrum, LOC duration, PTA duration, and blood-based biomarkers contributed to a marked increase in measurement precision as compared other indicators.

Evaluations of differential item functioning (DIF) by age and sex revealed no practically important DIF (Supplementary Figure S3). Women were, on average, 0.31 *SD* lower on latent severity than men. As compared to individuals who were under 30 years old, those aged 30–49 were 0.10 *SD* higher on severity, and those age 50 and older were 0.18 *SD* higher on severity.

### Associations between GCS-based TBI severity groups, novel IRT-based severity scores, and functional outcomes

**Fig. 3:**
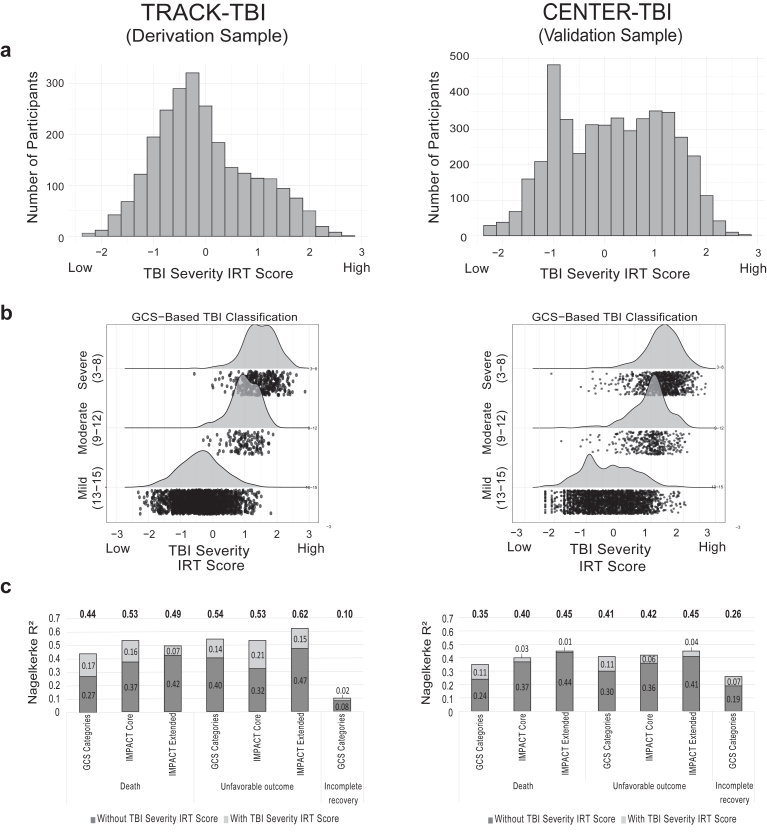
Distribution and prognostic value of item response theory (IRT)-based traumatic brain injury (TBI) severity scores. The model was developed from profiles of 24 indicators of acute TBI severity within the TRACK-TBI sample; those parameters were used to score individuals on the severity spectrum in both the TRACK-TBI (derivation) and CENTER-TBI (external validation) sample. Due to differences between studies, the CENTER-TBI subjects were scored using 17 of the original 24 variables. **(a)** Histogram depicting the distribution of TBI Severity IRT scores, which provides substantially more precision in estimating individual differences in TBI severity than traditional mild, moderate, and severe TBI classification (2446/2545 scores in the TRACK-TBI sample and 3438/4500 scores in the CENTER-TBI sample were unique). **(b)** Scatterplot of TBI Severity IRT scores versus traditional GCS-based classification of TBI; the robust, expected associations supports an interpretation that TBI Severity IRT scores reflect the same construct (TBI severity) as GCS-based classification, with more precision afforded by the IRT scores. Scatterplot points (individual subjects) are lagged in the direction of the y-axis to facilitate visualisation of the number of points along the x-axis. **(c)** Model Nagelkerke *R*^*2*^ for models predicting death, unfavourable outcome, and incomplete recovery at 6 months post-injury from traditional 3-level GCS-based TBI severity categories (mild, moderate, severe) and IMPACT model scores, as well as the increase in *R*^*2*^ observed after adding TBI Severity IRT scores to the models (all likelihood ratio test p ≤ 0.001). The figure illustrates that TBI Severity IRT scores, while not developed specifically to predict functional outcome, they nevertheless incrementally predict outcome beyond these other more traditional prognostic scores (GCS and IMPACT). *Abbreviations*: CENTER-TBI, Collaborative European NeuroTrauma Effectiveness Research in Traumatic Brain Injury study; GCS, Glasgow Coma Scale score; GOSE, Glasgow Outcome Scale-Extended; IMPACT, International Mission for Prognosis and Analysis of Clinical Trials in TBI; TRACK-TBI, Transforming Research and Clinical knowledge in TBI study.

Model-based estimates of TBI Severity IRT scores, scaled in *SD* units, were derived for each participant in both the TRACK-TBI and CENTER-TBI samples, using parameters estimated in the TRACK-TBI derivation sample. Leveraging the information contained within the 24 items (17 for CENTER-TBI) produced a score with much more granularity than classic mild, moderate, and severe TBI categories (see Fig. 3a), with 2446/2454 scores in the TRACK-TBI sample and 3438/4500 scores in the CENTER-TBI sample being unique. The monotonic relationship observed between the IRT scores and traditional GCS-based categories (Fig. 3b) supports that the two reflect the same construct. Supplementary Figure S4 shows the relationship between TRACK-TBI TBI Severity IRT scores and other classifications commonly used in the U.S. (the VA 3-group and the 4-group system described in Table 1).

Univariable logistic regression models demonstrated that TBI Severity IRT scores were robustly associated with discrete 6-month GOSE outcomes, especially death (*R*^2^ = 0.35–0.42) and unfavourable outcome (i.e., severe disability; *R*^2^ = 0.41–0.54; Supplementary Table S4). Moreover, in both samples TBI Severity IRT scores independently predicted all GOSE outcomes relative to GCS-based classifications (increase in *R*^2^ = 0.11–0.17, likelihood ratio test [LRT] p < 0.001), IMPACT Core (increase in *R*^2^ = 0.03–0.16, LRT p < 0.001), and IMPACT Extended scores (increase in *R*^2^ = 0.01–0.07, LRT p ≤ 0.001; Supplementary Table S5; Fig. 3c). Similar to the univariable models, gains in predictive power were especially strong when predicting death and unfavourable outcome. Models predicting death and incomplete recovery fit somewhat better in the TRACK-TBI sample, whereas models predicting incomplete recovery fit better in the CENTER-TBI sample (e.g., univariable model predicting outcome from IRT scores *R*^2^ = 0.24 versus 0.08).

Finally, a sensitivity analysis examined the performance of TBI Severity IRT scores in TRACK-TBI computed without blood-based biomarkers. Scores computed with and without biomarkers were correlated *r* = 0.97 (Supplementary Figure S5). Prognostic models incorporating these recomputed IRT scores yielded the same overall pattern of results (i.e., independent prediction of TBI Severity IRT scores computed without biomarkers), with model *R*^*2*^ values lower by, on average, *R*^*2*^ = 0.03 relative to those that used biomarkers in IRT score calculations (Supplementary Table S6).

## Discussion

In the large (*N* = 2545) TRACK-TBI sample of Level I trauma centre patients aged 17 years and older with GCS 3–15 TBI, we used IRT to model the continuum of TBI severity from 24 clinical, head CT, and blood-based biomarker features assessable soon after injury. We identified four key findings. First, a well-fitting one-factor model provided empirical support for the widespread assumption that TBI severity exists on a continuum. This continuum can be indexed across sex and age groups by GCS components, pupil reactivity, duration of altered consciousness (LOC, PTA), and objective CT and blood-based biomarkers. Second, IRT information curves provided a novel view of the level of TBI severity indexed by each indicator and the relative ability of the indicators to characterise individuals’ positions along the severity continuum. Third, blood-based biomarkers collected within 24 h of injury—particularly GFAP—proved useful in indexing the entire injury continuum. Finally, we demonstrated the validity of IRT-based TBI severity scores by showing that these were associated with traditional GCS-based severity categories and incrementally improved prediction of death and injury-related disability beyond GCS-based severity categories and IMPACT scores, within both the TRACK-TBI sample and the CENTER-TBI sample. That the model yielded robust, prognostic TBI Severity IRT scores within each of these samples is particularly remarkable in light of differences across studies in health systems and the fact that CENTER-TBI IRT scores were derived from a subset of information available (17/24 variables) in the TRACK-TBI sample.

Our study addresses the calls of expert working groups assembled by the National Institute of Neurological Disorders and Stroke (NINDS) to develop new approaches for characterising TBI severity.^11^^,^^16^ A 2007 working group recommended that new methods ideally would integrate clinical effects of injury (e.g., GCS) with more objective markers.^11^ Since then, significant progress has been made in collecting large prospective TBI samples needed to act on these recommendations to validate blood-based biomarkers for clinical decision making. Our results are responsive to the 2007 working group's recommendation in this regard as well as the 2024 initiative's emphasis on characterising TBI from a combination of clinical, head CT, and blood-based biomarker indicators.^16^ This study aligns well with these consensus-based initiatives, while illustrating a rigorous scientific method that can refine evolving TBI conceptualisations.

Our results also contribute to growing evidence that incorporating blood-based biomarkers improves the characterisation of TBI severity.^47^ For example, our findings suggest that GFAP, already FDA-cleared for assisting clinical neuroimaging decisions,^20^ also contributes to differentiating persons on the severity spectrum. This was especially valuable in the lower half of the severity spectrum, where other indicators (e.g., GCS, CT) are less useful. For persons in the upper half of the severity spectrum, biomarkers combined with clinical observations could be invaluable in settings where CT is unavailable to guide decisions about the need and urgency for transfer to a facility with neuroimaging and neurosurgical services. Two other widely-used TBI biomarkers, UCH-L1 and S100B, improved characterisation of severity but less so than GFAP. That these three biomarkers were most informative is understandable given their higher specificity to TBI relative to NSE and hsCRP and their robust associations with other indicator variables, especially CT findings.^48^, ^49^, ^50^, ^51^ However, because these markers vary in their half-lives but were sampled at a single timepoint, we caution against interpreting their relative performance. More generally there is a need for standardised approaches to biomarker sampling and analyses in order to realise their full potential as indicators of severity.

This new method of scoring TBI severity yields many opportunities for advancing the clinical assessment and management of TBI. Adopting the TBI Severity IRT score in practice would promote recognition of the wide individual differences of TBI, addressing aforementioned concerns of patient harm due to traditional TBI labels and staging. Moreover, TBI Severity IRT scores could support tailored interventions, more accurate prognoses, and improved clinical management. They also could be integrated into electronic health records, enabling automatic, multimodal data-driven injury assessments, providing added clinical information based on multimodal data to support injury characterisation and offer opportunities for tailored, evidence-based management decisions. For instance, scores might help stratify patients into follow-up care pathways suited to their severity. For example, to manage healthcare resources it may be appropriate to funnel lower-severity injuries to primary care for monitoring, provide active outreach and nurse case management for more severe injuries, and offer specialised multidisciplinary rehabilitation routinely to individuals above a certain severity level. As compared to traditional, imprecise TBI classification systems, the increased precision and sophistication of this novel IRT-based approach to grading severity affords greater opportunity to develop evidence-based approaches to characterise and manage diverse TBIs.

Another advantage of the present approach is its ability to score individuals on TBI severity even when there is incomplete data, as was illustrated in our application of the model to the CENTER-TBI sample where only 17/24 original indicators were available. With further validation, this method would allow clinicians in diverse settings and with different resources to score injuries on a unified index. Mobile scoring tools could be deployed to further increase accessibility in diverse settings, while offering feedback about the accuracy of estimates based on the data available as well as interpretive feedback. For example, in a field/pre-hospital setting, clinical signs and point-of-care blood-based biomarkers like GFAP may be used to estimate injury severity and aid in decisions about evacuation or transfer for further care. Hospital systems with comprehensive resources could incorporate all indicators from the IRT model to enable precise positioning of patients along the severity continuum, or to add new information to that gathered in the pre-hospital setting, affording more precise estimation of severity. These capabilities would also be of value in research, providing a way to index and compare TBI severity among samples assessed with different TBI severity indicators, as is common across sport, civilian, and military TBI studies.

Following conventions in grading TBI severity, our model is mainly based on information available on the first day of injury (with the exception of LOC and PTA duration). Future work could investigate whether adding other acute indicators (e.g., hypotension, hypoxia, lesion location),^24^ or variables reflecting patients’ evolving clinical course, operates to improve severity characteriaation.^47^ Future work should also cross-validate the model in new samples, extend the model to other TBI subpopulations (such as persons with injuries that do not necessitate treatment at Level I trauma centres), and solicit clinician feedback to explore strategies for future clinical implementation. Work to promote clinical implementation might benefit from examples from other areas of medicine that routinely quantify clinically relevant measures (e.g., bone density, regional brain volume) on a continuous scale and provide interpretable output for patients and clinicians.^52^^,^^53^ Finally, simplified versions of the model could be developed to balance precision and accessibility.

A strength of the current study is that analyses included the full TRACK-TBI sample to derive the model, a feature enabled by limited missingness on most variables and the use of full-information methods for IRT parameter estimation. Given expert recommendations to incorporate objective (e.g., radiographic, biochemical) signs of TBI into measurement models of TBI severity,^11^ it was valuable to initiate this line of inquiry using a large Level I trauma-centre based sample, as this provided sufficient variability in specific CT findings to estimate relations between these findings and the continuum of TBI. Additionally, the TRACK-TBI sample comprised a large number of individuals with more subtle TBI as reflected in these characteristics (e.g., *N* = 471 with GCS 15 CT− TBI). The resulting model assigned a unique TBI score to 2446/2545 individuals in the sample, highlighting the ability of the IRT model to differentiate a wide spectrum of TBI severity.

Although the study samples are fairly representative of adult TBI cases that require prompt ED evaluation, they are not representative of all TBI. Thus, the findings should not be generalised to other TBI subpopulations, such as sport-related concussion, which is typically managed without emergency department evaluation. Additionally, although we confirmed the model can be applied across sex and age groups, future studies should verify model fit in important subgroups such persons varying in peripheral injury severity,^54^, ^55^, ^56^, ^57^, ^58^, ^59^ which may influence the validity and interpretation of blood-based biomarkers and other model components. Work of this kind would serve as a valuable next step toward clinical implementation of the IRT-based staging model introduced here. Beyond generalisability, another limitation of this study is that certain indicators may not have been optimally collected. Blood, for example, was collected at one timepoint within 24 h of injury (12 h or later for many samples), often missing the early peak of UCH-L1 and S100B and preceding the peak of hsCRP.^35^ The relative information provided by the blood-based biomarkers should be interpreted with this limitation in mind. Additionally, the TRACK-TBI study did not assess LOC and PTA duration through standardised assessments or verify reliability of these data, which raises the possibility of that these clinical signs could be more informative if assessed under more controlled conditions. Finally, these indicators were missing more often than other model variables, which may increase the possibility of estimation bias particularly if data were MNAR.^60^ Although most model indicators had limited missingness and were plausibly assumed to be MCAR or MAR, MNAR cannot be verified in real datasets and is therefore a potential threat to validity for any study.^61^ While techniques to estimate the impact of potential MNAR are emerging, they require making assumptions that cannot be verified and are not well validated for IRT models.^62^^,^^63^

Unlike IMPACT scores, which were developed to predict functional outcome and, in turn, provide an early estimate of TBI severity, our IRT-based scores were developed by modelling a latent dimension underlying covariation among observed TBI indicators and estimating the relationship between those indicators and the latent dimension. This may explain why we did not uniformly see reduced prognostic modelling performance in the external validation sample. The finding that IRT-based severity scores incrementally predicted functional outcomes when combined with IMPACT scores suggests that IRT methods can complement the more traditional regression-based approach to developing indices that inform prognosis and conceptualisation of TBI.

In summary, the current study fills a need to grade TBI severity using indicators from multiple measurement domains (clinical, neuroimaging, blood-based biomarkers)^11^ and provides an initial demonstration that IRT—a quantitative tool widely used in other fields to develop practical clinical assessments^64^, ^65^, ^66^—provides a powerful and interpretable method to develop evidence-based strategies to characterise TBI. This study also provides a flexible model for integrating diverse data into a unified model of TBI severity, a model that could be adapted to integrate other relevant measures in the future. This rigorous, clinically relevant approach to scoring TBI severity addresses longstanding challenges in TBI classification and lays a strong foundation for continued innovation and progress in the field.

## Contributors

All authors read and approved the final manuscript. Authors LDN and BEM both had access to and verified the data and findings for this manuscript. **LDN**: Conceptualisation, Methodology, Data Curation, Formal analysis, Validation, Writing-Original Draft, Visualisation, Funding acquisition; **BEM**: Methodology, Formal analysis, Writing-Review & Editing; **ER**: Methodology, Writing-Review & Editing; **JKY**: Methodology, Writing-Review & Editing; **SB**: Methodology, Writing-Original Draft; **CJP**: Methodology, Writing-Review & Editing; **NT**: Writing-Review & Editing; **ELY**: Data Curation, Writing-Review & Editing; **RDA**: Project administration, Writing-Review & Editing; **AIRM**: Funding acquisition, Supervision, Writing-Review & Editing; **DKM**: Funding acquisition, Supervision, Writing-Review & Editing; **LW**: Supervision, Writing-Review & Editing; **GTM**: Funding acquisition, Conceptualisation, Supervision, Writing-Review & Editing; **TRACK-TBI Investigators**: Investigation, Writing-Review & Editing. **CENTER-TBI Participants and Investigators**: Investigation, Writing-Review & Editing.

## Data sharing statement

A de-identified version of the TRACK-TBI data used in this manuscript, alongside a data dictionary and analysis scripts, have been uploaded to the odc-tbi (odc-tbi.org) data repository platform, an NIH-supported repository where data are made available for FAIR reuse under a CC -BY 4.0 (attribution) licence. Data reuse requires full attribution of the dataset authors by citing: Nelson L. D. (2024). TRACK-TBI Acute IRT Data, Open Data Commons for Traumatic Brain Injury, ODC-TBI: https://odc-tbi.org/data/1168.

The TRACK-TBI study protocol and data collection forms is publicly available at the following website: https://tracktbi.ucsf.edu/researchers.

The CENTER-TBI data sharing policy is available at https://www.center-tbi.eu/data. CENTER-TBI dataset version 3.1 Opal was used in this manuscript.

## Declaration of interests

Some of the blood-based biomarker measurements for the TRACK-TBI study were performed in kind by Abbott Laboratories.

**LDN**: Received salary support for unrelated research from the U.S. Department of Defense, Centers for Disease Control and Prevention, and Medical College of Wisconsin Advancing a Healthier Wisconsin Endowment; personal compensation for independent consulting unrelated to this work for Resolys Bio, Inc.; and served as a chair for the Psychosocial and Environmental Modifiers Working Group for the 2024 NINDS TBI Classification and Nomenclature Initiative.). **NT**: Received salary support for unrelated research from the U.S. federal government.

**RDA**: Received support for other research from the U.S. National Institutes of Health and Department of Defense; in-kind contributions from MesoScale Discoveries for immunoassay kits and reagents for unrelated research; and stock options and service on professional advisory boards for BrainBox Solutions, LLC and Nia Therapeutics. **GTM**: Received salary support for work on the TRACK-TBI study from the National Institute of Neurological Disorders and Stroke (NINDS); support for other research from the NINDS, Department of Defense/MTEC, Abbott Laboratories, National Football League; Funding from OneMind for patient engagement; and served on the Steering Committee for the 2024 NINDS TBI Classification and Nomenclature Initiative. **AIRM**: Received consulting fees from NeuroTrauma Sciences. **LW**: Received consulting fees from NeurotraumaSciences, Mass General Brigham and University of Wisconsin.

**DKM**: Was supported by the CENTER-TBI grant (EU FP7 No 602150) and by funding for UK TBI-Repository and Data Portal Enabling Discovery (TBI-REPORTER) Grant (Ref: MR/Y008502/1), and is in receipt of research support, consultancy and/or lecture fees from NeuroTrauma Sciences, Lantamannen AB, GlaxoSmithKline Ltd, PressuraNeuro Ltd; Dompe; Invex Ltd; Abbot Ltd; and Integra Neurosciences Ltd).

## Appendix A Supplementary data

Study Group
