# Supplementary material for: Beyond mild, moderate, and severe traumatic brain injury: modelling severity from clinical, neuroimaging, and blood-based indicators
Source: eBioMedicine. 2025 Nov 4;121:106001. doi: 10.1016/j.ebiom.2025.106001 (PMC12637077; doi:10.1016/j.ebiom.2025.106001)
Supplement: Supplementary Figures and Tables [file mmc2.docx]

**Supplementary Material**

Nelson, LD, Magnus, BE, Yue, JK, Balsis, S, Patrick, CJ, Temkin, N, Yuh, EL, Diaz-Arrastia, R, Ryu, E., Maas, A.I.R., Menon, D.K., Wilson, L., Manley, GT, & the TRACK-TBI Investigators, on behalf of the CENTER-TBI Participants and Investigators. Beyond mild, moderate, and severe traumatic brain injury: Modeling severity from clinical, neuroimaging, and blood-based indicators. *eBioMedicine,* 106001, doi: 10.1016.j.ebiom.2025.106001*.*

**Table of Contents**

| **Supplementary Table s1.** Variables included in acute TBI severity IRT model  **Supplementary Figure s1.** Scree plot from exploratory factor analysis (EFA) model of 24 TBI indicators in the TRACK-TBI sample.  **Supplementary Table s2.** Item factor loadings from exploratory factor analysis model in the TRACK-TBI sample.  **Supplementary Table s3**. Item response theory model parameters (TRACK-TBI derivation sample).  **Supplementary Figure s2.** Item information curves from a single item response theory (IRT) model of 24 TBI severity indicators  **Supplementary Figure s3.** Differential item functioning (DIF) analysis showing no meaningful DIF of the item response theory model by age and sex.  **Supplementary Figure s4**. Scatterplots depicting the association between TBI Severity IRT scores and other TBI severity classification schemes (TRACK-TBI sample).  **Supplementary Table s4.** Univariable logistic regression models predicting 6-month functional outcome using GCS-based classification of TBI (mild, moderate, or severe), IMPACT scores, or TBI severity IRT scores (TRACK-TBI and CENTER-TBI samples).  **Supplementary Table s5**. Incremental predictive value of acute TBI severity IRT scores as compared to GCS-based classification of TBI severity and IMPACT scores for predicting 6-month functional outcomes (TRACK-TBI and CENTER-TBI samples).  **Supplementary Figure s5**. Scatterplot depicting the association between TBI Severity IRT scores calculated with and without blood-based biomarkers (TRACK-TBI sample).  **Supplementary Table s6**. Sensitivity analysis examining the independent predictive value of TBI Severity IRT scores on 6-month functional outcomes, where IRT scores were computed without blood-based biomarkers (TRACK-TBI sample). | **Page**  **2**  **3**  **4**  **5**  **7**  **8**  **9**  **10**  **12**  **13**  **14** |
| --- | --- |

**Supplementary Table s1.** Variables included in acute TBI severity IRT model

|  | TRACK-TBI Derivation Sample | CENTER-TBI Validation Sample |
| --- | --- | --- |
| **Head CT^1^** |  |  |
| Subarachnoid hemorrhage | X | X |
| Acute subdural hemorrhage | X | X |
| Skull fracture | X | X |
| Contusion | X | X |
| Edema | X |  |
| Downward herniation | X |  |
| Midline shift | X | X |
| Shear | X | X |
| Epidural hematoma | X | X |
| Upward cerebellar herniation | X |  |
| Intraventricular hemorrhage | X | X |
| Duret hemorrhage | X |  |
| Extraaxial hematoma | X | X |
| **Clinical signs** |  |  |
| Admission GCS eye | X | X |
| Admission GCS verbal | X | X |
| Admission GCS motor | X | X |
| Nonreactive pupils | X | X |
| LOC duration | X |  |
| PTA duration | X |  |
| **Blood-based biomarkers^1^** |  |  |
| GFAP | X | X |
| UCH-L1 | X | X |
| NSE | X | X |
| S100B | X | X |
| hsCRP | X |  |

*Note*. N=24 indicators of TBI severity used to establish item response theory (IRT) model parameters in the TRACK-TBI sample. Collaborators with content area and study expertise reviewed the variables for availability and harmonization with the CENTER-TBI study sample, and selected 17 variables with sufficient equivalence to score CENTER-TBI study participants on the latent TBI severity dimension, using IRT parameters established in the TRACK-TBI derivation sample.

*Abbreviations*. CT = computed tomography; CENTER-TBI, Collaborative European NeuroTrauma Effectiveness Research in Traumatic Brain Injury (CENTER-TBI); GCS = Glasgow Coma Scale; GFAP = glial fibrillary acidic protein; hsCRP = high-sensitivity C-reactive protein; NSE = neuron-specific enolase; S100b = S100B calcium-binding protein B; TRACK-TBI, Transforming Research and Clinical Knowledge in Traumatic Brain Injury; UCH-L1 = ubiquitin C-terminal hydrolase L1

^1^CT and blood-based biomarkers collected < 24 hours of injury

**Supplementary Figure s1.** Scree plot from exploratory factor analysis model of 24 TBI indicators in the TRACK-TBI sample. The ratio of the first to second eigenvalue was of highest interest, as an indication of sufficient unidimensionality for item response theory modeling to yield relatively unbiased parameter estimates. In this case, the ratio was 6.0, well over the recommended cutoff of > 4, and fit statistics were good for the 1-factor model (root mean square error of approximation = .08, comparative fit index = .96, Tucker Lewis Index = .96).


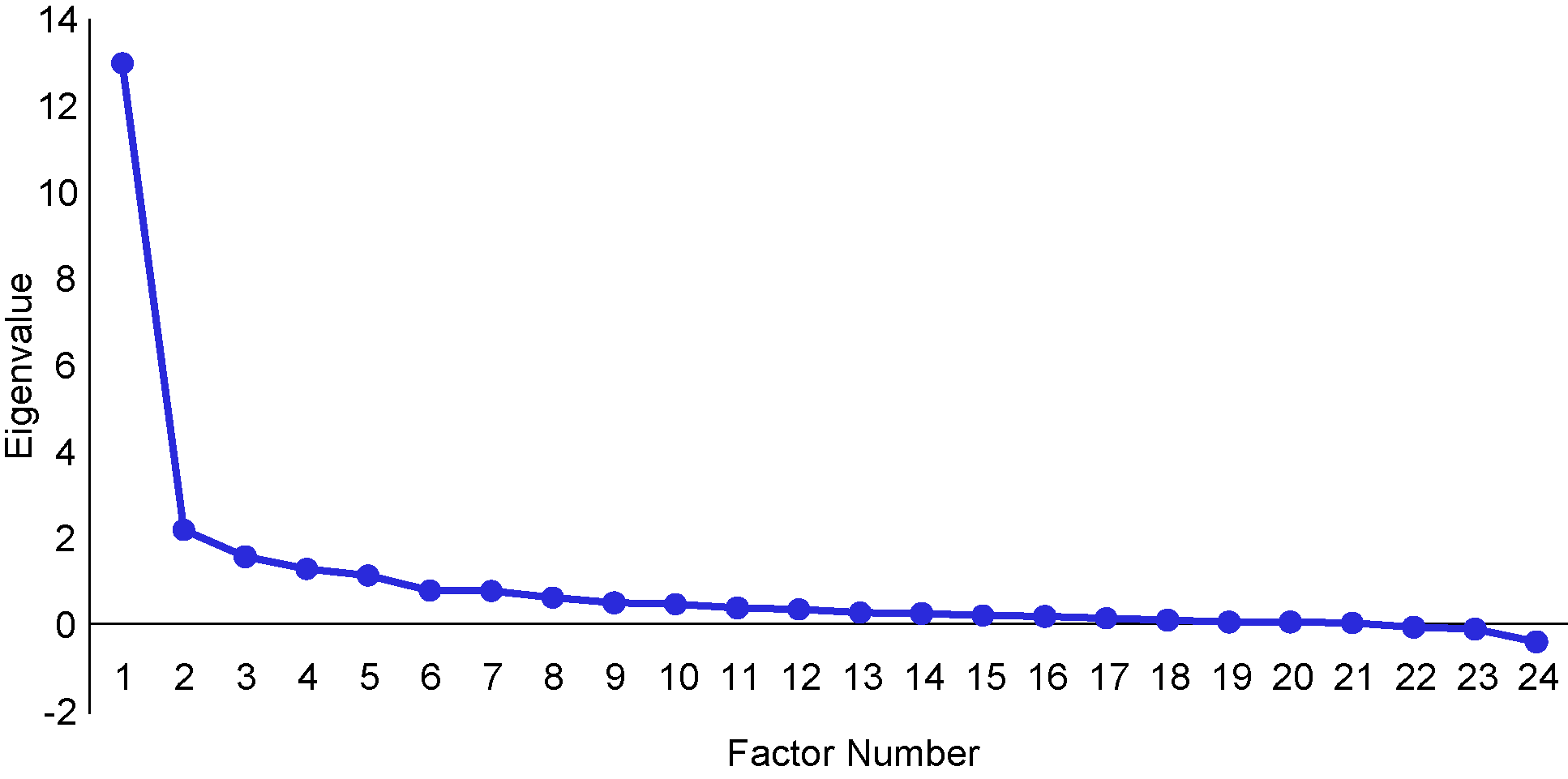


**Supplementary Table s2.** Item loadings from exploratory factor analysis model of traumatic brain injury severity indicators in the TRACK-TBI sample.

|  | Loading |
| --- | --- |
| **Head CT^1^** |  |
| Subarachnoid hemorrhage | ·82 |
| Acute subdural hemorrhage | ·82 |
| Skull fracture | ·83 |
| Contusion | ·84 |
| Edema | ·90 |
| Downward herniation | ·93 |
| Midline shift | ·89 |
| Shear | ·53 |
| Epidural hematoma | ·66 |
| Upward cerebellar herniation | ·79 |
| Intraventricular hemorrhage | ·54 |
| Duret hemorrhage | ·83 |
| Extraaxial hematoma | ·44 |
| **Clinical signs** |  |
| Admission GCS eye | ·96 |
| Admission GCS verbal | ·91 |
| Admission GCS motor | ·99 |
| Nonreactive pupils | ·82 |
| LOC duration | ·69 |
| PTA duration | ·62 |
| **Blood-based biomarkers^1^** |  |
| GFAP | ·78 |
| UCH-L1 | ·73 |
| NSE | ·53 |
| S100B | ·72 |
| hsCRP | ·49 |

*Note*. CT = computed tomography; GCS = Glasgow Coma Scale; GFAP = glial fibrillary acidic protein; hsCRP = high-sensitivity C-reactive protein; NSE = neuron-specific enolase; S100b = S100B calcium-binding protein B; TRACK-TBI, Transforming Research and Clinical Knowledge in Traumatic Brain Injury; UCH-L1 = ubiquitin C-terminal hydrolase L1

^1^Collected < 24 hours of injury.

**Supplementary Table s3**. Item response theory model parameters (TRACK-TBI derivation sample)

|  | *a* | *b1* | *b2* | *b3* | *b4* | *b5* | *b6* | *b7* | *b8* | *b9* |
| --- | --- | --- | --- | --- | --- | --- | --- | --- | --- | --- |
| Head CT^1^ |  |  |  |  |  |  |  |  |  |  |
| Subarachnoid hemorrhage | 2·18 | 0·39 |  |  |  |  |  |  |  |  |
| Acute subdural hemorrhage | 2·00 | 0·72 |  |  |  |  |  |  |  |  |
| Skull fracture | 1·92 | 0·85 |  |  |  |  |  |  |  |  |
| Contusion | 2·10 | 0·97 |  |  |  |  |  |  |  |  |
| Edema | 3·06 | 1·42 |  |  |  |  |  |  |  |  |
| Downward herniation | 3·64 | 1·57 |  |  |  |  |  |  |  |  |
| Midline shift | 2·75 | 1·59 |  |  |  |  |  |  |  |  |
| Shear | 1·14 | 2·26 |  |  |  |  |  |  |  |  |
| Epidural hematoma | 1·22 | 2·35 |  |  |  |  |  |  |  |  |
| Upward cerebellar herniation | 3·74 | 2·55 |  |  |  |  |  |  |  |  |
| Intraventricular hemorrhage | 1·28 | 2·82 |  |  |  |  |  |  |  |  |
| Duret hemorrhage | 3·16 | 3·11 |  |  |  |  |  |  |  |  |
| Extraaxial hematoma | 0·98 | 3·51 |  |  |  |  |  |  |  |  |
| Clinical signs |  |  |  |  |  |  |  |  |  |  |
| Admission GCS eye | 3·09 | 0·85 | 1·12 | 1·23 |  |  |  |  |  |  |
| Admission GCS verbal | 2·60 | 0·34 | 1·16 | 1·26 | 1·53 |  |  |  |  |  |
| Admission GCS motor | 3·33 | 0·94 | 1·26 | 1·44 | 1·49 | 1·57 |  |  |  |  |
| Nonreactive pupils | 2·41 | 1·96 | 2·17 |  |  |  |  |  |  |  |
| LOC duration | 1·46 | -1·64 | -0·79 | 1·19 | 1·45 | 2·05 | 2·85 |  |  |  |
| PTA duration | 1·51 | -1·24 | -1·01 | 0·00 | 0·29 | 1·37 | 2·06 |  |  |  |
| Blood-based biomarkers^2^ |  |  |  |  |  |  |  |  |  |  |
| GFAP | 2·59 | -1·52 | -1·03 | -0·67 | -0·37 | -0·07 | 0·24 | 0·59 | 1·02 | 1·60 |
| UCH-L1 | 1·66 | -1·82 | -1·20 | -0·76 | -0·39 | -0·04 | 0·32 | 0·73 | 1·22 | 1·92 |
| NSE | 1·05 | -2·48 | -1·62 | -1·02 | -0·51 | -0·02 | 0·47 | 1·00 | 1·62 | 2·51 |
| S100B | 1·62 | -1·86 | -1·23 | -0·79 | -0·41 | -0·06 | 0·31 | 0·71 | 1·21 | 1·91 |
| hsCRP | 1·13 | -2·37 | -1·56 | -0·99 | -0·52 | -0·08 | 0·36 | 0·84 | 1·42 | 2·28 |

*Note*. *a* reflects item discrimination; *b* values reflect thresholds (i.e., severity along the TBI continuum). For a variable with x categories, there are (x – 1) thresholds reflecting each step from a lower category to a higher category. For example, the verbal clinical symptoms on the GCS are coded in one variable with 5 categories (0 vs 1, 2, 3, 4). This 5-category variable has 5 – 1 = 4 b parameters. The first b parameter, b1, equals 0·35, meaning that 0·35 SDs of TBI severity are required before a person has a 50% likelihood of scoring higher than 0. The second b parameter, b2, equals 1·17, meaning that 1·17 SDs of TBI severity are required before a person has a 50% likelihood of scoring higher than 1, and so on. *Abbreviations*: CT = computed tomography; GCS = Glasgow Coma Scale; GFAP = glial fibrillary acidic protein; hsCRP = high-sensitivity C-reactive protein; NSE = neuron-specific enolase; S100B = S100 calcium-binding protein B; TRACK-TBI, Transforming Research and Clinical Knowledge in Traumatic Brain Injury; UCH-L1 = ubiquitin C-terminal hydrolase L1

^1^Head CT findings reflect acute findings from neuroimaging performed < 24 hours of injury. ^2^Blood-based biomarkers categorized into 6–11 equally sized subgroups for item response theory modeling.

**Supplementary Figure s2. Item information curves from a single item response theory (IRT) model of 24 TBI severity indicators, stratified by measurement domain for readability**. The figure reflects the combination of Figure 1 a, b, and c to illustrate that the data represent a single model combining neuroimaging, clinical, and blood-based biomarker indicators of TBI severity. The x-axis reflects the latent TBI severity spectrum modeled from the associations between the indicators using IRT. The y-axis reflects IRT *information*, which reflects the precision with which each variable can be used to measure individuals on the severity dimension, which can vary at different levels of severity. Higher information reflects lower standard errors to estimate individuals at a given level of severity. *Abbreviations*: CT, computed tomography; EDH, epidural hematoma; GCS, Glasgow Coma Scale; GFAP, glial fibrillary acidic protein; hsCRP, high-sensitivity C-reactive protein; IVH, intraventricular hemorrhage; NSE, neuron-specific enolase; LOC, loss of consciousness; PTA, posttraumatic amnesia; S100B, S100 calcium binding protein B; SAH, subarachnoid hemorrhage; SDH, subdural hematoma; TBI, traumatic brain injury; UCH-L1, ubiquitin C-terminal hydrolase

**
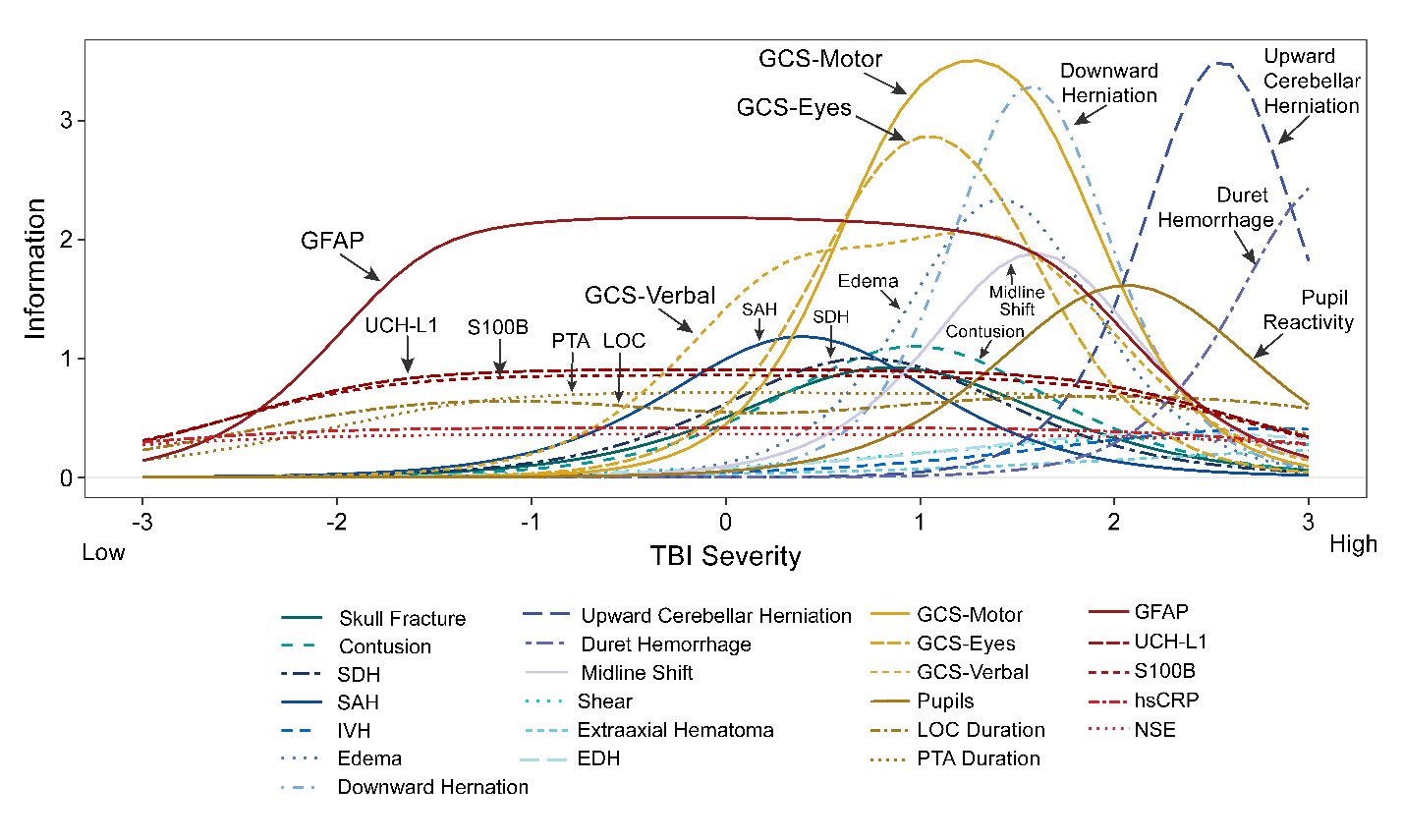
**

**Supplementary Figure s3. Differential item functioning (DIF) analysis showing no meaningful DIF of the item response theory model by age and sex**. Within the model developed from 24 indicators in the TRACK-TBI sample, DIF was tested using sex (male, female) and age (< 30, 30-49, and 50+ years old) as categorical variables. Each variable was tested using a series of Wald tests to evaluate both discrimination and threshold DIF, initially treating all other items as anchors. Items found to be DIF-free were sequentially constrained to equality across groups until no additional items could be identified as DIF-free. For analyses of sex, 0/24 items showed discrimination DIF; 5/24 items exhibited statistically significant threshold DIF applying a Benjamini-Hochberg correction to control the false discovery rate. For analyses of age, 5/24 items showed statistically significant discrimination DIF; 11/24 items showed threshold DIF. Further investigation found these statistically significant differences to have negligible practical impact. To illustrate this, we generated expected total score functions using group-specific item parameter estimates for the items flagged for DIF, while using common item parameter estimates for the DIF-free items. As shown in the figure below, the expected total scores as a function of latent TBI severity (theta) were nearly identical across groups for both sex and age. These results suggest that the detected DIF had minimal practical impact on the scale’s overall measurement properties and would not meaningfully affect substantive conclusions drawn from the scores.
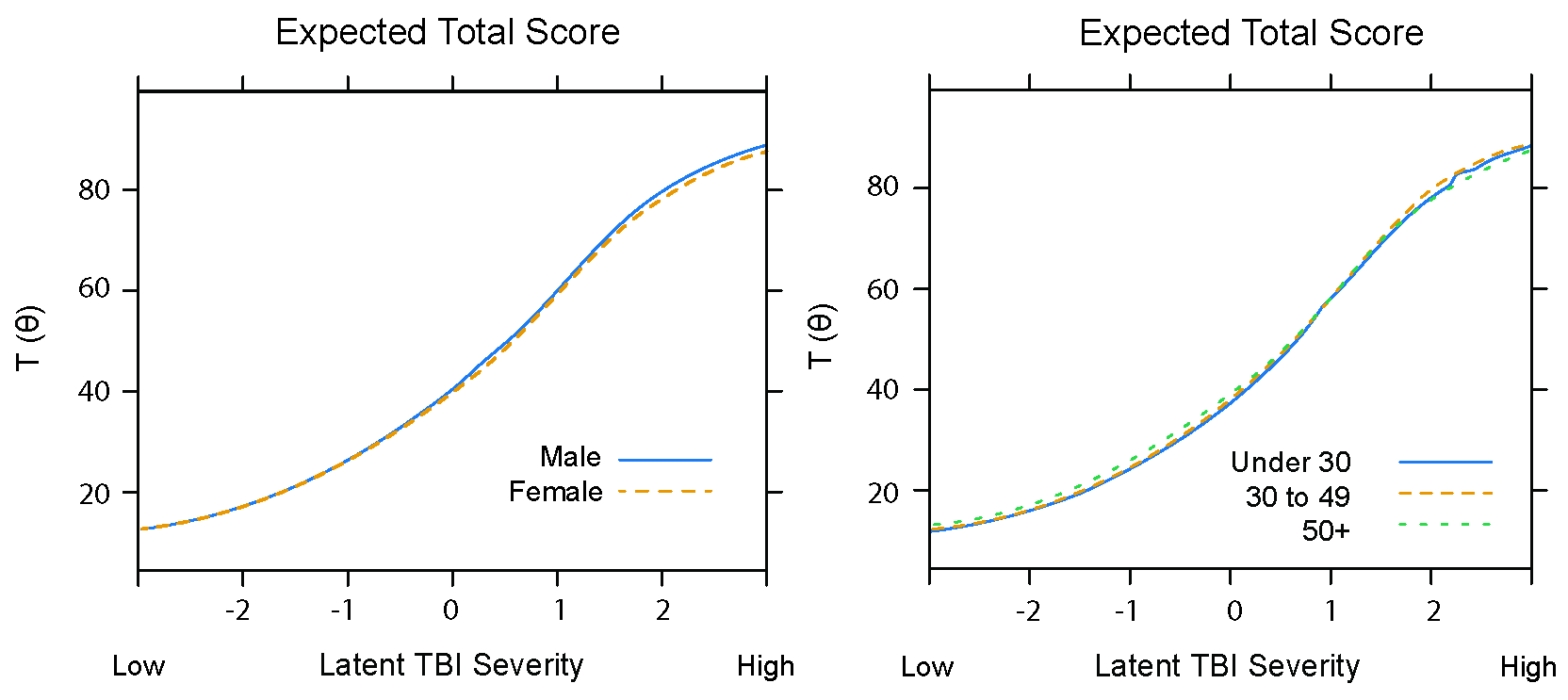


**Supplementary Figure s4**. Scatterplots depicting the association between TBI Severity IRT scores (TRACK-TBI Derivation Sample) and other TBI severity classification schemes, namely the system used by the U.S. Department of Veterans Affairs/Department of Defense (left) and a 4-group classification system commonly used in the field of neuropsychology (see **Table 1** in the main manuscript for more details). Points (individual subjects) are lagged in the direction of the y-axis to facilitate visualization of the number of points along the x-axis. *Abbreviations*: c-MTBI, complicated mild traumatic brain injury; TBI, traumatic brain injury; u-MTBI, uncomplicated mild traumatic brain injury


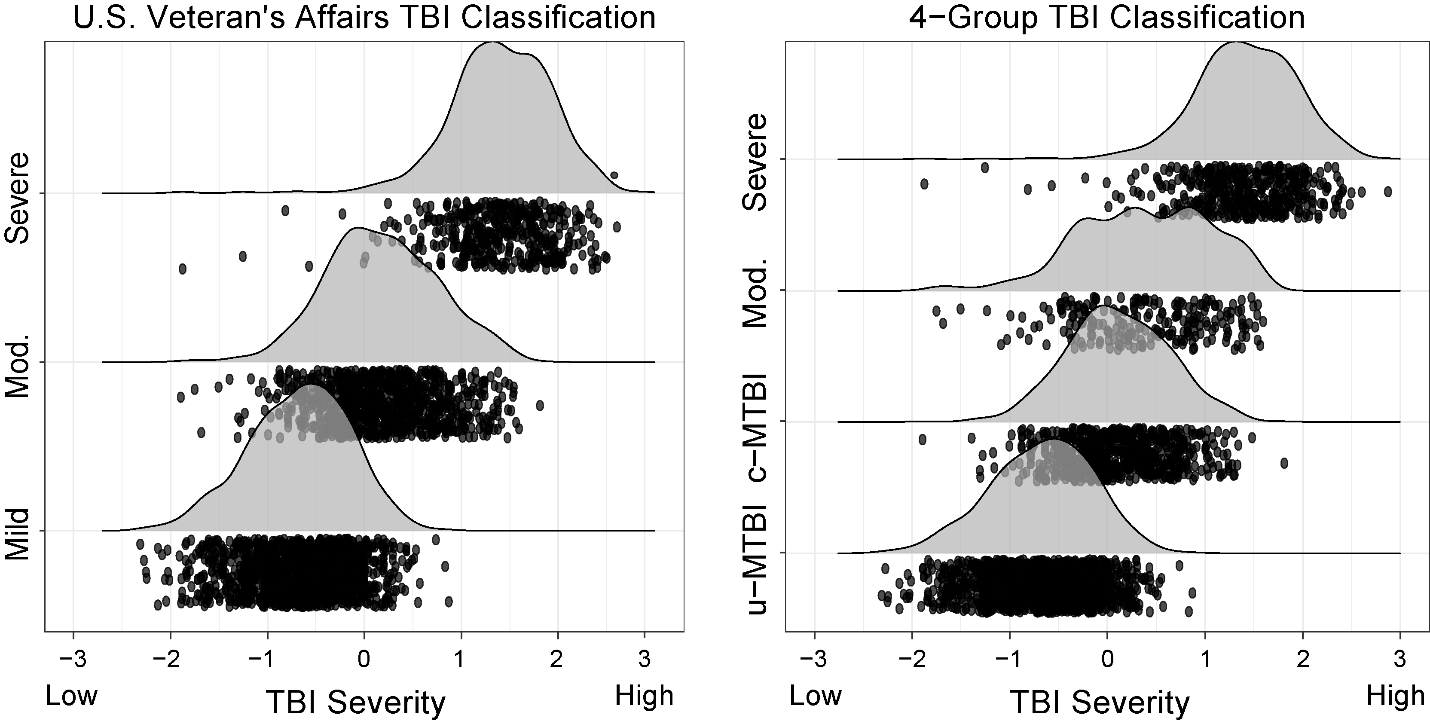


**Supplementary Table s4. Univariable logistic regression models predicting 6-month functional outcome using GCS-based classification of TBI (mild, moderate, or severe), IMPACT scores, or TBI severity IRT scores (TRACK-TBI and CENTER-TBI samples)**

|  | **TRACK-TBI Derivation Sample** | | **CENTER-TBI Validation Sample** | |
| --- | --- | --- | --- | --- |
| **Death** | **OR (95% CI)** | **Nagelkerke R^2^** | **OR (95% CI)** | **Nagelkerke R^2^** |
| GCS |  | ·27 |  | ·24 |
| GCS 9-12 vs. GCS 13-15 | 6·84 (2·92, 14·75) |  | 7·42 (5·28, 10·40) |  |
| GCS 3-8 vs. GCS 13-15 | 19·41 (12·24, 31·77) |  | 11·67 (9·06, 15·17) |  |
| IMPACT Core | 3·94 (2·82, 5·73) | ·37 | 3·47 (2·95, 4·13) | ·37 |
| IMPACT Extended score | 4·15 (2·79, 6·59) | ·42 | 3·61 (3·05, 4·32) | ·44 |
| TBI Severity IRT score | 8·84 (6·38, 12·62) | ·42 | 5·70 (4·81, 6·81) | ·35 |
| **Unfavorable Outcome** |  |  |  |  |
| GCS |  | ·40 |  | ·30 |
| GCS 9-12 vs. GCS 13-15 | 13·88 (7·59, 25·03) |  | 5·99 (4·46, 8·02) |  |
| GCS 3-8 vs. GCS 13-15 | 32·41 (21·90, 49·06) |  | 13·13 (10·61, 16·31) |  |
| IMPACT Core | 3·02 (2·32, 4·04) | ·32 | 3·17 (2·73, 3·72) | ·36 |
| IMPACT Extended score | 4·09 (2·89, 6·09) | ·47 | 3·15 (2·71, 3·69) | ·41 |
| TBI Severity IRT score | 11·52 (8·62, 15·82) | ·54 | 5·74 (4·97, 6·68) | ·41 |
| **Incomplete Recovery** |  |  |  |  |
| GCS |  | ·08 |  | ·19 |
| GCS 9-12 vs. GCS 13-15 | 3·44 (1·84, 7·18) |  | 3·89 (2·87, 5·37) |  |
| GCS 3-8 vs. GCS 13-15 | 5·30 (3·53, 8·31) |  | 9·39 (7·24, 12·39) |  |
| TBI Severity IRT score | 1·83 (1·62, 2·08) | ·08 | 2·72 (2·49, 2·98) | ·24 |

*Note*. Model P all < ·001. Of N=1697 participants with a 6-month GOSE score, 125 died (GOSE 1), 248 had unfavorable outcome (GOSE < 5), and 1214 had incomplete recovery (GOSE < 8). *N*s for models incorporating GCS-based TBI categories were *N* = 1653 (based on availability of GCS total scores and 6-month GOSE outcome data). Models incorporating IMPACT scores included the subsample who were GCS < 13 and who had all IMPACT indicators available (Model *Ns* = 227 to 316). Model Ns using TBI Severity IRT scores were *N* = 1697. Outcomes reflect Glasgow Outcome Scale-Extended (GOSE) scores at 6 months post-injury of 1 (death), <5 (unfavorable outcome), and incomplete recovery (GOSE<8). *Abbreviations*: CENTER-TBI, Collaborative European NeuroTrauma Effectiveness Research in Traumatic Brain Injury study (validation sample); GCS, Glasgow Coma Scale; IRT, item response theory; TBI, traumatic brain injury; IMPACT, International Mission for Prognosis and Analysis of Clinical Trials in TBI; TRACK-TBI, Transforming Research and Clinical knowledge in TBI study (development sample)

**Supplementary Table s5**. Incremental predictive value of acute TBI severity IRT scores as compared to GCS-based classification of mild, moderate, or severe TBI (top) and IMPACT scores (bottom) for predicting 6-month functional outcomes (TRACK-TBI and CENTER-TBI samples).

| **First Predictor** | **Sample** | **Model 1: First Predictor Only** | **Model 2: Adding TBI Severity IRT Score** | | |
| --- | --- | --- | --- | --- | --- |
| *Death Outcome* |  | **Nagelkerke R^2^** | **Nagelkerke R^2^** | **Likelihood Ratio Test**  χ^2^(1) |  |
| GCS-Based 3-Group | TRACK-TBI | ·27 | ·44 | 124·57 (p < ·001) |  |
|  | CENTER-TBI | ·24 | ·35 | 234·50 (p < ·001) |  |
| IMPACT Core | TRACK-TBI | ·37 | ·53 | 48·22 (p < ·001) |  |
|  | CENTER-TBI | ·37 | ·40 | 30·76 (p < ·001) |  |
| IMPACT Extended score | TRACK-TBI | ·42 | ·49 | 13·56 (p < ·001) |  |
|  | CENTER-TBI | ·44 | ·45 | 10·32 (p = ·001) |  |
| *Unfavorable Outcome* |  |  |  |  |  |
| GCS-Based 3-Group | TRACK-TBI | ·40 | ·54 | 153·61 (p < ·001) |  |
|  | CENTER-TBI | ·30 | ·41 | 301·51 (p < ·001) |  |
| IMPACT Core | TRACK-TBI | ·32 | ·53 | 71·86 (p < ·001) |  |
|  | CENTER-TBI | ·36 | ·42 | 61·85 (p < ·001) |  |
| IMPACT Extended score | TRACK-TBI | ·47 | ·62 | 41·65 (p < ·001) |  |
|  | CENTER-TBI | ·41 | ·45 | 3·32 (p < ·001) |  |
| *Incomplete Recovery* |  |  |  |  |  |
| GCS-Based 3-Group | TRACK-TBI | ·08 | ·10 | 19·12 (p < ·001) |  |
|  | CENTER-TBI | ·19 | ·26 | 194·21 (p < ·001) |  |

*Note*. Outcomes reflect Glasgow Outcome Scale-Extended (GOSE) scores at 6 months post-injury of 1 (death), <5 (unfavorable outcome), and incomplete recovery (GOSE<8). Model Ns for models incorporating GCS-based TBI categories were *N* = 1653 (based on availability of 6-month GOSE outcome data). Models incorporating IMPACT scores included the subsample who were GCS < 13 and who had all IMPACT indicators available (Model *Ns* = 227 to 316). Outcomes reflect Glasgow Outcome Scale-Extended (GOSE) scores at 6 months post-injury of 1 (death), <5 (unfavorable outcome), and incomplete recovery (GOSE<8).

*Abbreviations*: CENTER-TBI, Collaborative European NeuroTrauma Effectiveness Research in Traumatic Brain Injury study (validation sample); GCS, Glasgow Coma Scale; IRT, item response theory; TBI, traumatic brain injury; IMPACT, International Mission for Prognosis and Analysis of Clinical Trials in TBI; TRACK-TBI, Transforming Research and Clinical knowledge in TBI study (derivation sample)

**Supplementary Figure s5**. Scatterplot depicting the association between traumatic brain injury (TBI) severity item response theory (IRT) scores calculated with (y-axis) and without (x-axis) blood-based biomarkers within the TRACK-TBI sample. While scores are highly correlated (r = 0.97), it is apparent that measurement error is higher in the lower half of the severity continuum, where there are other model indicators do not provide substantial information to distinguish persons on severity.


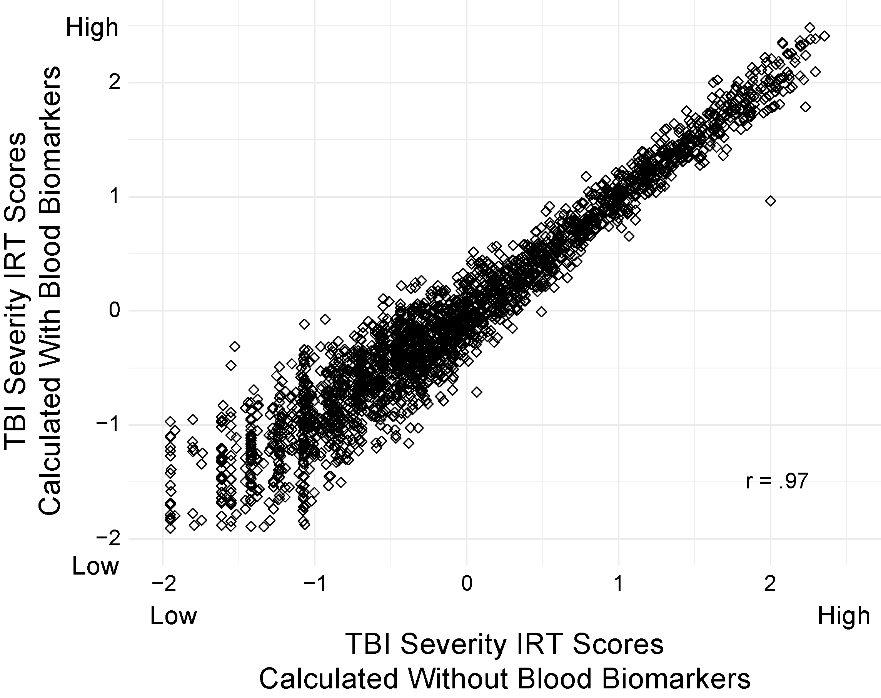


**Supplementary Table s6**. Sensitivity analysis examining the independent predictive value of TBI Severity IRT scores on 6-month functional outcomes within the TRACK-TBI sample, where IRT scores were computed without blood-based biomarkers. While overall model fit is slightly lower than was the case with scores computed with the blood-based biomarkers (e.g., Nagelkerke R^2^ for the death and unfavorable outcome models fell ·42–·38, ·54–·50, respectively), the TBI Severity IRT scores computed without biomarkers still independently predicted functional outcome, after controlling for traditional GCS-based categorization and IMPACT scores.

| **First Predictor** | **Model 1: First Predictor Only** | **Model 2: Adding TBI Severity IRT Score** | | |
| --- | --- | --- | --- | --- |
| **Death** | **Nagelkerke R^2^** | **Nagelkerke R^2^** | **Model fit** |  |
| GCS-Based 3-Group | ·27 | ·39 | χ^2^(1) = 85·64 (p < ·001) |  |
| IMPACT Core | ·38 | ·49 | χ^2^(1) = 34·27 (p < ·001) |  |
| IMPACT Extended score | ·43 | ·47 | χ^2^(1) = 8·80 (p = ·003) |  |
| TBI Severity IRT Score | ·38 | - | - |  |
| **Unfavorable Outcome** |  |  |  |  |
| GCS-Based 3-Group | ·40 | ·50 | χ^2^(1) = 113·65 (p < ·001) |  |
| IMPACT Core | ·32 | ·48 | χ^2^(1) = 54·25 (p < ·001) |  |
| IMPACT Extended score | ·47 | ·58 | χ^2^(1) = 28·52 (p < ·001) |  |
| TBI Severity IRT Score | ·50 | - | - |  |
| **Incomplete Recovery** |  |  |  |  |
| GCS-Based 3-Group | ·08 | ·09 | χ^2^(1) = 12·68 (p < ·001) |  |
| TBI Severity IRT Score | ·08 | - | - |  |

*Abbreviations*: GCS, Glasgow Coma Scale; IRT, item response theory; TBI, traumatic brain injury; IMPACT, International Mission for Prognosis and Analysis of Clinical Trials in TBI
